# Mr. Spencer Wells

**Published:** 1854-12

**Authors:** 


Mr. Spencer Wells' new work on *Gout*, has sweet consolation for those afflicted with this aristocratic disease. He says:—"Among the present members of the Houses of Parliament, those who are known to be subject to gout are among the most distinguished for an ancestry rendered illustrious by high thoughts and noble deeds, for their own keen intelligence, for the assistance they have afforded to improvements in arts, science, and agriculture, and for the manner in which they have led the spirit of the age. If it were proper to mention names, I believe I could prove this to be the case; and I never met with a real cause of gout, in other classes of the community, in a person not remarkable for mental activity, unless the tendency to gout was clearly inherited. It is perfectly true that butlers, hall-porters, and other individuals in like easy circumstances, are often subject to gout; but many such persons are the sons of parents who have lived in similar situations, and have received from their progenitors hereditary predisposition. I have seen a great many examples of the rule, and scarcely an exception, that when a person in an inferior situation is subject to gout, and one of his parents was not similarly effected he is a person of superior abilities or attainments."

We like that; and nothing so humiliates our pride as to look at our plebian toes, disgracefully and hereditarily innocent of any aristocratic tumor—not even a gentlemanly corn is there.—*Buffalo Med. Journal.*

